# Soft Denture Liner Adhesion to Conventional and CAD/CAM Processed Poly(Methyl Methacrylate) Acrylic Denture Resins-An In-Vitro Study

**DOI:** 10.3390/ma14216614

**Published:** 2021-11-03

**Authors:** Sara Mohammad Al Taweel, Hanan Nejer Al-Otaibi, Nawaf Labban, Afnan AlFouzan, Huda Al Shehri

**Affiliations:** Department of Prosthetic Dental Sciences, College of Dentistry, King Saud University, P.O. Box 60169, Riyadh 11545, Saudi Arabia; haalotaibi@ksu.edu.sa (H.N.A.-O.); nalabban@ksu.edu.sa (N.L.); aalfouzan@ksu.edu.sa (A.A.); hAlShehri@ksu.edu.sa (H.A.S.)

**Keywords:** acrylic resins, air-particle abrasion, CAD/CAM technologies, soft liners, tensile bond strength

## Abstract

This study aimed to evaluate the airborne-particle abrasion surface treatment effects on the tensile bond strength (TBS) between resilient denture liner and CAD/CAM or conventional heat polymerized poly (methyl methacrylate) (PMMA) acrylic denture resins. A total of 48 dumbbell-shaped specimens (70 mm in total length, and 12 mm and 7 mm in diameter at the thickest and thinnest section, respectively) were prepared from CAD/CAM and conventional acrylic resins. Before relining with denture liner, 12 specimens from each material were surface-treated by 110 µm Al_2_O_3_ airborne-particle abrasion, and the remaining specimens served as control (no treatment). Following relining, all the specimens were aged by thermal cycling (1000 cycles, 5–55 °C). The TBS of denture liner to acrylic denture resins was tested in a universal testing apparatus at a 5 mm/min crosshead speed. The debonded surfaces were visually examined for the failure modes. ANOVA and multiple comparisons posthoc analysis tests were applied to determine the significant difference in TBS between the study groups (α = 0.05). A significant difference in TBS was observed between the control and surface treated groups (*p* < 0.001) for both acrylic resins materials. However, there was no statistically significant difference in bond strength between the acrylic resins materials (*p* = 0.739). Surface treatment with airborne-particle abrasion demonstrated increased TBS of the soft denture liners to acrylic resins. The TBS of conventional and CAD/CAM acrylic resins to soft denture liners were not considerably different.

## 1. Introduction

Long-term denture wear causes considerable changes in the supporting structures and can result in chronic pain and discomfort, particularly in the mandible [1]. The accelerated resorption of the alveolar ridges promotes the formation of a sharp and narrow alveolar ridge crest that exerts too much pressure. This causes severe problems for the patient and necessitates the need for a durable denture and ridge protection [2]. Accordingly, relining techniques to overcome the difficulties of ill-fitting dentures gain significant importance in such conditions [3]. Besides being non-invasive, the method is cost-effective compared to fabricating new dentures and provides comfort to the patient [4,5].

Denture liners are either classified as hard (often made of polymethylmethacrylate) or resilient [6]. The resilient liners are likewise classified as short-term and long-term liners. Short-term liners are intended to be worn for up to 30 days, whereas long-term denture liners maintain their resiliency for longer than 30 days and can be worn for up to a year [7,8,9]. Resilient denture lining materials are preferred over hard denture liners because of their cushioning effect on the fitting surface of the dental prostheses to promote a more uniform force delivery, relieve confined pressure, and enhance the prosthesis retention by engaging soft tissue undercuts [4]. On the contrary, resilient denture liners have significant drawbacks, such as surface flaws and porosity, residual palatableness after use, odor and water uptake, color instability, difficulty in maintaining hygiene, and premature thickening due to solubilization of the plasticizer [7]. The adhesion failure between the liner and the acrylic resins is a serious concern with resilient denture liners [10,11]. The durable bond strength between the denture liners and acrylic resins is recommended to prevent interfacial detachment at the borders of the denture. Additionally, the adhesive failure between the acrylic resins and the soft liners facilitates bacterial growth and expedite the disintegration of the soft lining material [12,13]. Previous studies have suggested using different techniques to enhance the adhesion of resilient liners to the denture base. These include the introduction of surface roughness to denture base by airborne-particle abrasion, chemical etching, acrylic bur roughening, and lasing [8,14,15,16,17,18].

The advancement of computer-aided design and manufacturing (CAD/CAM) technologies has fundamentally changed the approach to denture processing in recent years [19,20,21]. The dentures have been fabricated using CAD/CAM technology since the 1990s; nevertheless, due to the lack of scientific evidence, they are still regarded as a relatively new approach [22]. CAD-CAM-fabricated dentures have improved material characteristics and reduced treatment visits, saving patients and clinicians time and less cost. It is simple to access and fabricate new dentures if they’re damaged or lost as the data is digitally stored [23,24].

The bonding properties of resilient denture liners and conventionally fabricated denture base polymers have been extensively investigated [3,7,8,14,25,26,27,28,29]. The use of CAD/CAM technology in clinical dental practice is increasing day by day. However, the scientific evidence regarding the bonding characteristics of denture liners to CAD/CAM fabricated denture resins is limited [3]. The nature of the adhesion between soft liners and technologically advanced CAD/CAM acrylic materials will provide valuable input to clinicians regarding their routine application in clinical practice. Additionally, the surface treatment of CAD/CAM dentures by airborne-particle abrasion surface treatment in promoting adhesion between the denture base and soft denture liners also needs to be thoroughly investigated.

Therefore, this laboratory study reports the findings of the airborne-particle abrasion surface treated or non-surface treated CAD/CAM PMMA denture materials in promoting the adhesion between the soft denture liners and denture base. Furthermore, these findings will be compared with surface treated or non-surface treated conventional heat-polymerized PMMA denture base materials as inadequate bonding reveals the severe flaws of denture base materials in the clinical environment. The null hypothesis tested was that airborne-particle abrasion would have no effect on TBS and that the bond strength of resilient denture liners would be unaffected by the type of denture base material used.

## 2. Materials and Methods

**Figure 1:**
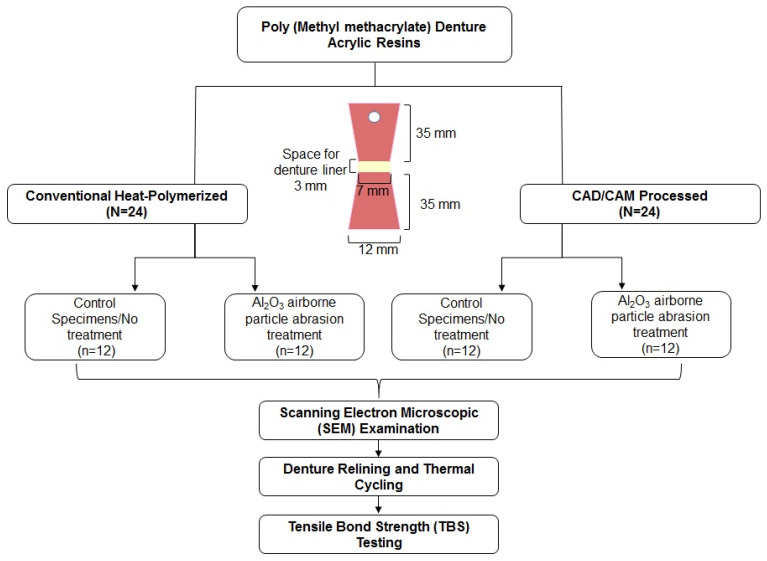
Specimen distribution and study procedure.

The flow chart illustrating the specimen distribution and study procedure is presented in Figure 1.

### 2.1. Specimen Preparation

A total of 48 dumbbell-shaped specimens (70 mm in total length, and 12 mm and 7 mm in diameter at the thickest and thinnest section, respectively) were prepared from conventional heat polymerized PMMA resin (Heat-cured, Major. Base.20, Moncalieri, Italy) and CAD/CAM (Opera system, Principauté de Monaco, Monaco) PMMA resin blocks. The specimen design was in accordance with a previously published study [30]. A dumbbell-shaped brass pattern with a 3-mm thick and 7-mm diameter spacer at the center of the brass pattern was invested using gypsum (Moldabaster S, Heraeus Kulzer GmBH, Hanau, Germany) in a dental flask. The spacer determines the recommended thickness (3 mm) of the soft denture liners and allows the packing of the liner materials during the relining procedure in in-vitro conditions. According to the manufacturers’ recommendations, the conventional PMMA denture resin was processed and packed into the mold with the spacer intact. The processed resin was polymerized in a heat-curing unit at 70 °C for 90 min and elevated to 100 °C for another 30 min. After polymerization, the specimen was removed from the mold, the excess resin was trimmed, and the bonding surfaces were manually smoothened using 200-grit silicon carbide paper.

The CAD/CAM specimen was designed using CAD software (Zenotec^®^, Wieland Digital Denture, Ivoclar Vivadent, Schaan, Liechtenstein), and CAD/CAM blocks were milled using Zenotec^®^ selection (Ivoclar Vivadent, Schaan, Liechtenstein). The CAD/CAM specimens were finished similarly to the conventional PMMA specimens. The specimen dimension was confirmed using an electronic caliper with tolerance values of ±0.05 mm to the specified measurements. Then, a 5 mm hole was drilled 5 mm away from one end of the specimen using a benchtop electric drill press. This hole allowed the clamping of the specimen to the jig of the universal testing apparatus to ensure alignment of the specimens during the TBS test. All the specimens were handled by a single operator (S.M.A) for standardization.

### 2.2. Surface Treatment of Specimens and Scanning Electron Microscopic (SEM) Evaluation

All the specimens were stored in distilled water (37 ± 1 °C) to allow water saturation of the denture base polymer. Before surface treatment, the specimens were air-dried for 24 h and were randomly allocated into two groups (*n* = 12). One group served as control (PMMA-C, CAD/CAM-C) with no surface treatment, and the other group (PMMA-S, CAD/CAM-S) was treated by airborne-particle abrasion. The specimens were airborne particle abraded using Al_2_O_3_ particles (110 µm, 2.8 bar, 10 s) directed towards the specimen surface from a distance of approximately 10 mm, in a circular motion by an intra-oral air abrasion device (Danville Inc., San Ramon, CA, USA). The specimens were rinsed under running water and were air-dried to establish a remnant-free bonding interface.

A representative specimen from each material group was selected and examined under an SEM (Zeiss EVO LS 10; Carl-Zeiss-Strasse 22, Oberkochen, Germany) to demonstrate the surface modification after airborne particle abrasion. The acrylic specimens were mounted onto the aluminum supports using dual-side adhesive tape and gold sputter-coated in a vacuum. The SEM was operated at 20 kV in a vacuum and ×1000 magnification.

### 2.3. Denture Relining and Thermal Cycling Procedure

**Figure 2:**
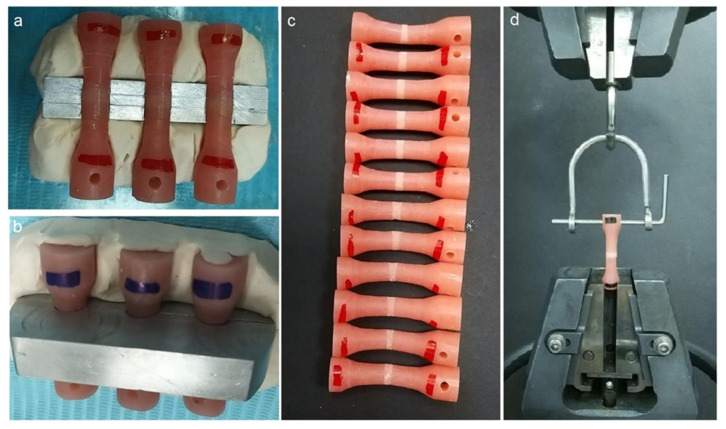
Specimen preparation and bond strength testing. (**a**) Specimen placed in the customized mold before soft liner application, (**b**) COE-SOFT liner allowed to polymerize in the mold, (**c**) completed specimen before thermal cycling, and (**d**) specimen placed in the jig of universal testing machine.

A customized metal mold with two indentation lines was used to ensure the 3-mm thickness of the denture relining material (Figure 2a). The resilient, auto-polymerizing reline material (COE-SOFT, GC America Inc., Alsip, IL, USA) was processed following the manufacturer’s recommendations. The reline material was packed and allowed to polymerize within the metal mold (Figure 2b). COE-SOFT polymerizes in approximately 15 min, but the setting time was extended for an additional one hour, accounting for a total polymerization time of 1 h and 15 min. The specimens (Figure 2c) were then subjected to 1000 thermal cycles (Huber 1100, SD Mechatronik GmbH, Germany) in a water bath (5–55 °C), a dwell time of 30 s and a transfer time of 15 s [21].

### 2.4. Tensile Bond Strength (TBS) Testing

The specimens were oriented vertically and secured to the lower grip fixture of a universal testing apparatus (Instron Corporation, Norwood, MA, USA) with a 0.5 kN load cell for the TBS test. The upper part of the specimen was clamped to the customized jig of the testing machine (Figure 2d). The test specimens were subjected to a tensile force with a constant velocity of 5 mm/min till failure. The maximum force required to demonstrate debonding of the specimens was recorded, and the TBS values were calculated using the below Formula (1):

(1) $$S=\frac{F}{A},$$

where S is the TBS values in megapascal (MPa), *F* is the maximum force in Newton (N), and *A* is the cross-sectional bonding area in mm^2^.

The fracture mode was determined by visually inspecting the debonded specimens. The failures were classified as either adhesive (complete partition at the interface between the denture resin and soft liner), or cohesive failure (cracking and damage within the soft liner material), or a combination of adhesive and cohesive failure (mixed failures).

### 2.5. Statistical Analysis

The data analysis was performed with IBM SPSS software (v.20, IBM Corp, Armonk, NY, USA). The mean and standard deviations were calculated for each material group; results were submitted to the one-way analysis of variance (ANOVA) followed by Tukey’s posthoc multiple comparison tests (α = 0.05).

## 3. Results

### 3.1. Scanning Electron Microscopic (SEM) Observation

**Figure 3:**
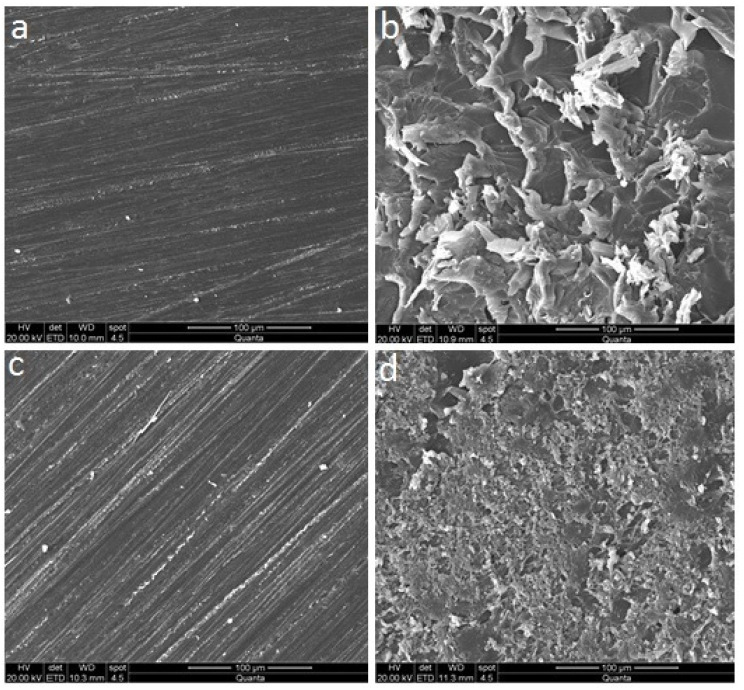
Scanning electron microscopy micrographs of the control and surface treated specimen groups before relining; (**a**) PMMA-C, (**b**) PMMA-S, (**c**) CAD/CAM-C, and (**d**) CAD/CAM-S.

The SEM micrographs of the control and surface treated representative specimen surfaces are presented in Figure 3. The SEM evaluation of surface-treated PMMA and CAD/CAM specimens (Figure 3b,d, respectively) showed significant surface flaws compared to their respective control groups (Figure 3a,c, respectively). However, the surface changes were more evident in the PMMA-S specimen (Figure 3b).

### 3.2. Tensile Bond Strength (TBS) Test Outcome

**Figure 4:**
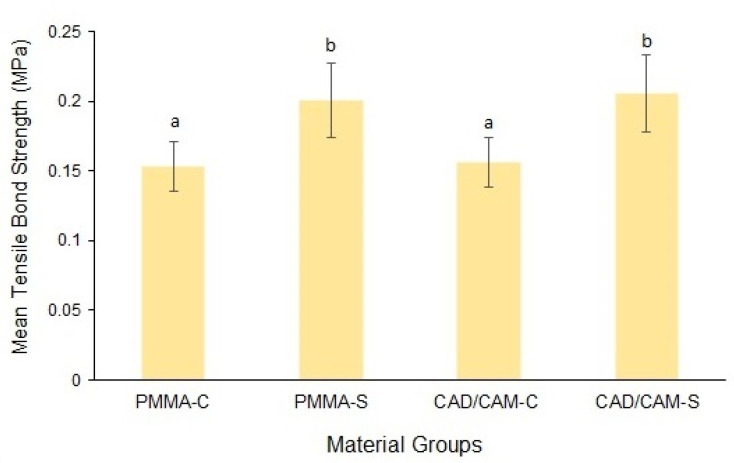
Mean tensile bond strength of the material groups. Bars indicate standard deviation.

The mean and standard deviation of the TBS values of the groups are presented in Figure 4. The mean TBS of the conventional PMMA was significantly higher in the surface treated group (0.2 ± 0.026) compared to the control group (0.15 ± 0.017) (*p* < 0.001). Similarly, the CAD/CAM material also demonstrated significantly higher bond strength values for the surface-treated specimens (0.21 ± 0.027) as compared to the control group (0.16 ± 0.017) (*p* < 0.001). The mean difference in the TBS values between the study materials was non-significant for both control (*p* = 0.739) and surface-treated groups (*p* = 0.634).

**Post-hoc interpretation:** Same lower case alphabet between the groups shows the non-significant difference between the groups. 

### 3.3. Failure Mode Analysis

The failure modes distribution of the specimens is presented in Table 1. Mixed failure (73%) was the predominant type among all the material groups, followed by adhesive failure (25%). A single instance of cohesive failure was observed in the CAD/CAM-S group (2%). The failure mode distribution was similar for all the groups, thus inferring that failure type was not dependent on surface modification.

## 4. Discussion

The current study evaluated the effect of airborne-particle abrasion surface treatment on the TBS between resilient denture liner and conventional heat polymerized or CAD/CAM PMMA based acrylic denture resins. The study’s outcome revealed that the airborne-particle abraded denture surface demonstrated significantly higher bond strength than their respective control groups (*p* < 0.001). However, comparing the bond strength values between the conventional and CAD/CAM processed PMMA demonstrated a non-significant difference for both control (*p* = 0.739) and surface-treated groups (*p* = 0.634). Therefore, the null hypothesis that the airborne-particle abrasion would have no effect on TBS and that the bond strength of denture liners would be unaffected by the type of denture base material used was partially accepted.

The bond strength of the soft liner to the acrylic denture is determined using the conventional peel, shear, and tensile testing methods. Peel and shear test are rarely used because of the difficulties in interpreting the test results. On the contrary, the tensile bond strength test is extensively used following the recommendation by ASTM and ISO (ISO 10139-2:2016) [31]. However, these laboratory tests do not precisely reflect the substantial bond strength of soft liners to the denture base under a clinical environment. During each of these tests, the material is only exposed to one form of stress instead of simultaneous exposure to varying degrees of cyclic masticatory forces acting in ambiguous oral conditions [32].

The clinical performance of the soft denture liner depends on a strong bond between the two components [21]. The major reason for bond failure could be related to the main structural differences in the chemical composition of the liner and the denture base, as well as the lack of chemical interactions between them [30,33]. Two PMMA denture base polymers with similar chemical compositions were chosen in this study; however, they were processed either by conventional or CAD/CAM methods. It has been demonstrated that processing methods affect the surface properties of the denture base materials and consequently the bonding to the soft liners [34]. Based on these assumptions, it was expected that CAD/CAM processed denture base resins would produce significantly higher bond strength compared to conventional PMMA resins.

On the contrary, the current study results demonstrated a non-significant difference in adhesion strength between the CAD/CAM processed and conventional PMMA materials. This may be related to the conventional group’s strict heat polymerization based on the manufacturer’s recommendation, which could have enhanced its physical properties. Furthermore, the standardized polishing methods applied in this study could also have contributed to the non-significant differences in bond strength between the study materials. Similarly, Choi et al. [3] evaluated the TBS of heat-polymerized, CAD/CAM and auto-polymerized PMMA denture base resins to three different resilient denture liners. The authors reported that CAD/CAM processed acrylic resins had the lowest TBS among the tested PMMA materials.

The adhesion strength of resilient liners to denture base resins can be enhanced via chemical surface treatment (acetone, isobutyl methacrylate, methyl formate-methyl acetate, phosphoric acid-etching), tribo-chemical silica coating, Nd: YAG and Er: YAG laser application, oxygen plasma treatment, and primer or monomer application [7,29]. The bond strength values of the liners on a roughened denture surface are almost twice as compared to a smooth surface [35]. The surface irregularities facilitate the flow of liners, thereby creating a mechanical interlock of the liners to the denture base [36].

The use of airborne-particle abrasion of the denture surface to enhance the adhesion strength has found conflicting results. Few authors reported a decrease in bond strength between the soft liners and the acrylic denture following airborne-particle abrasion [8,37,38,39]. The authors stated that surface roughening by air particle abrasion was not adequate to allow the flow of the liner to the surface irregularities. On the contrary, Usumez et al. [18] showed that airborne-particle abrasion of the PMMA resins before liner application showed higher mean TBS than the control group. Similarly, Nakhaei et al. [16] reported that airborne-particle abrasion of PMMA resins with 110 µm alumina enhances the adhesion between resilient liners and denture base. These conflicting results between studies could be due to the differences in the acrylic resins and soft-liners used, different sizes of abrasive particles, and the pressure [18]. This was further substantiated by the findings of Akin et al. [40], who reported that 120 µm Al_2_O_3_ particles are the best particle size to enhance bond strength compared to 50 µm, 60 µm, and 250 µm Al_2_O_3_ particles. The outcome of the present study is in agreement with those studies [16,18] reporting an increased bond strength following airborne-particle abrasion.

In the oral cavity, dentures are exposed to foods with different temperatures. The fluctuations in temperatures may significantly impact the adhesion of the liner-denture base interface due to the consumption of hot food and cold beverages. In simulating such an environment, the specimens were subjected to aging by thermal cycling. Previous studies have evaluated the thermal cycling effect on the bond strength of soft denture liners to conventional PMMA resins [3,10,41]. The bonding between soft liners and denture base materials is altered by aging in the water bath, the PMMA resin’s structure, and the water bath’s temperature. Following water immersion, the plasticizer from the soft liners is leached out, and water absorption occurs, affecting the dentures’ durability and dimensional stability. This will lead to the brittleness of denture liners, thus transferring the external load to the liner-denture base resin interface [10]. Furthermore, the bond strength diminishes with increased aging time due to the swelling and altered rigidity of the lining materials, as well as the production of fissures and internal stress at the interface between liners and denture surface. Although certain materials’ bond strengths diminish as a result of internal stress at the denture-liner interface, the bond strengths of others remain stable or may improve as the cross-linking process continues [30]. The materials in this study were subjected to 1000 cycles corresponding to one year of clinical service [21]. This was deemed adequate for the study because durable denture liners are shown to have a maximum lifespan of one year [42].

The soft, resilient liners should possess bond strength values greater than 0.44 MPa to denture bases to be acceptable for the clinical situation, and a soft liner thickness of 2 to 3 mm to serve the purpose [29,35]. In the present study, all the study groups with a 3 mm thick, soft denture liner demonstrated values below the clinically acceptable limit. It is noteworthy that the bond strength values obtained in this study were after thermal cycling. The absence of the baseline bond strength values is the primary limitation of the present study. Our study’s main aim was to compare CAD/CAM and conventional PMMA materials and evaluate the effect of airborne-particle abrasion on the bond strength after aging. The in-vitro results could vary significantly from the ambiguous oral environment because of the various factors that could play a role, including denture hygiene, dietary habits, and masticatory forces. The inability to completely simulate the oral environment in the present study could be considered another limitation.

While reporting the present study findings, the authors were unaware of any in-vivo studies evaluating the bond strength. Therefore, the outcome of the in-vivo studies should be verified with the existing in-vitro results. In-vivo research evaluating the bond strength of soft liners to denture base resins should be prioritized in the future. The effect of different aluminum oxide particle sizes on TBS should be investigated further. In addition, there is a choice of CAD-CAM and 3D-printed denture base acrylic resins on the market that could be used in future studies.

## 5. Conclusions

The TBS of conventional and CAD/CAM PMMA acrylic resins to auto-polymerizing soft denture liners did not differ significantly. Surface treatment of denture base resin by 110 µm Al_2_O_3_-particle abrasion was effective in increasing the TBS of both materials. The bond strength of the specimens following thermal cycling in both material groups was below the clinically acceptable limit.

## Figures and Tables

**Table 1:** Failure mode distribution of the specimens.

Groups (*n* = 12)	Adhesive Failure	Cohesive Failure	Mixed Failure
PMMA-C	3	0	9
PMMA-S	4	0	8
CAD/CAM-C	2	0	10
CAD/CAM-S	3	1	8
% distribution	25%	2%	73%

## Data Availability

Data sharing is not applicable to this article.
